# Antiproliferative and Antibacterial Activities of* Cirsium scabrum* from Tunisia

**DOI:** 10.1155/2017/7247016

**Published:** 2017-07-12

**Authors:** Ramla Sahli, Céline Rivière, Cédric Dufloer, Claire Beaufay, Christel Neut, Joanne Bero, Thierry Hennebelle, Vincent Roumy, Riadh Ksouri, Joelle Quetin-Leclercq, Sevser Sahpaz

**Affiliations:** ^1^Institut Charles Viollette (EA 7394), Université de Lille, 59000 Lille, France; ^2^The Laboratory of Aromatic and Medicinal Plants, Biotechnology Centre of Borj-Cédria (CBBC), Hammam-lif, Tunisia; ^3^Pharmacognosy Research Group, Louvain Drug Research Institute, Université Catholique de Louvain, Avenue E. Mounier, No. 72, B01.72.03-1200 Brussels, Belgium; ^4^UDSL, INSERM U995, UFR Pharmacie, 59000 Lille, France

## Abstract

Several* Cirsium* species are known for their uses in traditional medicine and consequently are studied for their phytochemical content and their biological activities. In the framework of a previous study conducted on eight extremophile plants from Tunisia, we highlighted that the crude methanolic extract of* C. scabrum*, a not investigated thistle, showed moderate but quite selective cytotoxic activity against the cancerous cell line J774 compared to the noncancerous cell line WI38 (IC_50_ = 11.53 *μ*g/ml on J774, IC_50_ = 29.89 *µ*g/ml on WI38, and selectivity index = 2.6). In the current study, the partitions of the leaves of* C. scabrum* were analyzed for their antiproliferative activity on the same cell lines. From the most active petroleum ether partition, we isolated four triterpenoids including lupeol, taraxasterol acetate, and a (1 : 1) mixture of 25-hydroperoxycycloart-23-en-3*β*-ol and 24-hydroperoxycycloart-25-en-3*β*-ol. These two cycloartane-type triterpenoids are mostly responsible for this cytotoxic activity. On the other hand, the antimicrobial potential of this plant was also evaluated against 36 microorganisms. The moderate antibacterial activity against 6* Staphylococcus aureus* and 2* Dermabacter hominis* strains is mainly attributed to the butanol partition whose major compounds are glycosides of flavones.

## 1. Introduction

The genus* Cirsium* (thistle), belonging to the Asteraceae family, is well distributed around the world. About 200* Cirsium* species are distributed in Europe, North Africa, Asia, and North and Central America [1]. In agriculture,* Cirsium* species are often considered as invasive weeds against which massive means of control are deployed. By contrast, some species are easily cultivated by communities in dry steppes of Mongolia where fruits and vegetables are not easily afforded [2]. In some countries, such as in Turkey, some* Cirsium* species are considered as edible plants [3]. Different parts can be consumed, such as stems, roots, and dethroned young leaves [4]. Some species of the genus* Cirsium* are also used as medicinal plants in the traditional medicine of several countries. In some European countries, the tea prepared from the leaves of some* Cirsium *species possesses therapeutic virtues. It is the case in Central Italy where the decoction of the leaves of* Cirsium arvense* is used to treat abdominal pains and intestinal disturbances. The species is also frequently used as an emergency haemostatic for wounds [5]. The roots or the entire plants of more than ten species are used in China for the treatment of various ailments including hemorrhaging, jaundice, and gastrointestinal disorders [6]. According to literature, these traditional uses led to the consideration of the phytochemistry and the biological activities of some species.* Cirsium* species contain a variety of natural products including flavonoids, phenolic acids, lignans, sesquiterpenoids, triterpenoids, sterols, alkaloids, acetylenes, polyacetylenes, hydrocarbons, and a few other compounds [7]. Flavonoids and their glycosides are the main secondary metabolites of* Cirsium* species [6]. The compounds isolated from* Cirsium* species and extracts show many different biological activities including antimicrobial, antioxidant, antidiabetic, anti-inflammatory, vasorelaxant, astringent, hepatoprotective, and anticancer activities [8].

*Cirsium scabrum* (Poir.) Bonnet & Barratte is a tall thistle native of the Mediterranean region [4]. To our knowledge, no reports on the uses of this plant in the traditional medicine, on its chemical composition, and on its biological activities have been provided in the literature so far. In a previous study conducted on eight extremophile plants from Tunisia, we highlighted that the crude methanolic extract of* C. scabrum* showed moderate but quite selective cytotoxic activity against J774 compared to WI38 (IC_50_ = 11.53 *μ*g/ml on J774, IC_50_ = 29.89 *µ*g/ml on WI38, and selectivity index = 2.6) [9]. In addition, the therapeutic uses attributed to several* Cirsium* species prompt us to explore in more depth the cytotoxic effect of the leaves of* C. scabrum* and to identify the compounds responsible for this activity using a bioguided fractionation approach. As several* Cirsium* species demonstrate an antimicrobial potential [7], we used the same approach to evaluate the antimicrobial activity of this species against a panel of 36 microorganisms and to identify the bioactive natural products.

## 2. Material and Method

### 2.1. General Experimental Procedures

For the extraction and fractionation, synthesis grade methanol (MeOH), petroleum ether (PE), methylene chloride (CH_2_Cl_2_), and cyclohexane were furnished by VWR chemicals (Fontenay-sous-Bois, France), whereas ethyl acetate (EtOAc) and n-heptane were obtained from Carlo Erba (Val-de-Reuil, France). Toluene was purchased from Fisher Scientific (Illkirch, France). Water (W) was bidistilled. All organic solvents for Centrifugal Partition Chromatography (CPC) purification were of High-Pressure Liquid Chromatography (HPLC) grade except for the n-heptane which was of synthesis grade (Carlo Erba, Val-de-Reuil, France). Ethyl acetate (EtOAc) and methanol (MeOH) were purchased from Fisher Scientific (Illkirch, France). Water was purified using Millipore Integral 5 (France) water purification system with a resistivity of not less than 18 MΩ·cm^−1^. The chloroform-d6 and MeOD for Nuclear Magnetic Resonance (NMR) experiments were obtained from Euriso-Top (Gif-sur-Yvette, France).

Analytical Thin Layer Chromatography (TLC) was performed on precoated silica gel 60 F (0.25 mm, Merck, Germany) and the spots were visualized under UV (254 and 365 nm) before being sprayed with a solution of sulfuric anisaldehyde and heating at 100°C for 10 min. Silica gel 60 (63–200 *µ*m, Macherey-Nagel, Germany) and silica gel 60H (<63 *µ*m, Macherey-Nagel, Germany) were used for column chromatography.

Analytical HPLC was carried out using a Shimadzu binary LC-10AS pump, a SCL-10A UV-visible detector, and a Vision HT Basic C18 (5 *μ*m, 4 mm i.d. × 250 mm) column (Grace, Epernon, France). Preparative HPLC was performed using a Shimadzu HPLC system equipped with a LC-20AP binary high-pressure pump, a SPD-M20A photodiode array detector, and a VisionHT Basic C18 (5 *μ*m, 22 mm i.d. × 250 mm) column. The mobile phase was composed of 0.01% formic acid in water (solvent A) and acetonitrile (ACN) (solvent B). For analytical HPLC analysis, 20 *µ*l of extract (10 mg/ml in MeOH) was injected at 1 ml/ml, whereas, for preparative HPLC analysis, 200 mg of extract solubilized in MeOH was injected at 14 ml/min. The wavelength used was 254 nm. For both analyses, the following gradient elution program was used: 10–25% B (0–15 min), 25–26.5% B (15–20 min), 26.5–34.75% B (20–25 min), 34.75% B (25–28 min), 34.75–35.12% B (28-29 min), 35.12% B (29–32 min), and 35.12–100% B (32–35 min).

CPC was performed using an Armen instrument 250 mL rotor (SCPC-250-L) provided by Armen instrument (Saint-Avé, France). CPC analyses were monitored using Shimadzu pump and detector.

NMR spectra were recorded on a Bruker DPX-500 spectrometer (^1^H and ^13^C NMR at 500 MHz), except for compound** 2** identified on a 600 MHz spectrometer. For triterpenoids, MS analyses were carried out using a Trace DSQ mass spectrometer (Thermo Finnigan) operating in the electron-impact mode. Samples were analyzed in a full-scan mode (100–700 amu) and were injected directly with a DEP probe. The data obtained were compared with those of the database NIST. Data acquisition and processing were performed with Xcalibur software. For flavonoids, HRMS analyses were carried out in negative mode with a range of* m/z* 100–1000, using a Thermo Fisher Scientific Exactive Orbitrap mass spectrometer equipped with an electrospray ion source. The vaporizer temperature of the source was set at 100°C, the nitrogen sheath gas at 10–20, and the auxiliary gas at 2–6 (arbitrary units).

### 2.2. Plant Material

*Cirsium scabrum* was collected in August 2013 in the North of Tunisia (Goubellat) and was identified at the Biotechnology Centre of Borj-Cédria by Dr. Abderrazak Smaoui. A voucher specimen (181) was deposited at the Herbarium of the Laboratory of Extremophile Plants at the Biotechnology Centre of Borj-Cédria.

### 2.3. Preparation of the Methanolic Crude Extract of* C. scabrum* Leaves

260.8 g of* C. scabrum* leaves was dried at 25°C for a week and then powdered. Extraction was carried out by maceration at room temperature (MeOH, 15 ml per g of powder, 3 × 48 h). After filtration, extract was dried in vacuum at 35°C and lyophilized to afford 22.17 g of crude methanolic extract (yield = 8.5%).

### 2.4. Fractionation of the Methanolic Crude Extract of* C. scabrum* Leaves

The crude methanolic extract of* C. scabrum* leaves (CS = 12 g) was suspended in water (20 ml) and partitioned with PE, CH_2_Cl_2_, EtOAc, and butanol (10 × 60 ml). Partitions were concentrated at 35°C under reduced pressure to give PE partition (CSPE = 0.84 g), CH_2_Cl_2_ partition (CSMC = 1.46 g), EtOAc partition (CSEA = 1.03 g), and butanol partition (CSBt = 2.72 g). The remaining aqueous partition was freeze-dried and lyophilized (CSW = 5.16 g).

### 2.5. Isolation of Compounds from the CSPE Partition

2 g of the CSPE partition (obtained after two successive extractions and fractionations) was subjected to column chromatography (CC) on silica gel 60 (63–200 *µ*m) using a gradient of heptane/EtOAc (100 : 0 to 70 : 30) to give seven fractions (CSPE1–CSPE7) based on their TLC behaviour (heptane/EtOAc, 7 : 3). Compound** 1** (21.9 mg) was isolated by this process (heptane/EtOAc, 95 : 5).

Compound** 2** (1.5 mg) was isolated from the combined fractions CSPE1 and CSPE2 (346.3 mg) by a silica gel 60H CC, eluted with PE/CH_2_Cl_2_ (25 : 5).

Preparative Centrifugal Partition Chromatography (CPC) was carried out to isolate compounds from the most active combined fractions CSPE4 and CSPE5. CPC separation was conducted using a quaternary biphasic solvent system: Arizona X (n-Hept/EtOAc/MeOH/H_2_O, 9 : 1 : 9 : 1). Ten quaternary biphasic solvent systems (Arizona) were tested in order to choose the optimal system [10]. The two phases (aqueous and organic) of Arizona X system were prepared. The rotor was entirely filled at 30 ml/min and 500 rpm with the aqueous stationary phase (400 mL) in the ascending mode. The rotation speed was increased to 1600 rpm and the organic mobile phase was pumped into the column in ascending mode at a flow-rate of 8 ml/min. Separation was performed using 400 mg of combined partitions CSPE4/CSPE5, in ascending mode for 25 min, and then switched to descendant mode for 27 min. Fractions of 8 ml were collected every min by a Gilson FC 204-fraction collector (52 subfractions: X1–X52). Fractions were checked by TLC developed with heptane/EtOAc (7 : 3). CPC analysis allowed the direct purification of a (1 : 1) mixture of compounds** 3a** and** 3b** (X30–X33: 9.6 mg).

### 2.6. Isolation of Compounds from the CSBt Partition

Major compounds of the CSBt partition (compounds** 4a**/**4b** and** 5**) were isolated by preparative HPLC using the following gradient elution program: 10–25% B (0–15 min), 25–26.5% B (15–20 min), 26.5-34.75% B (20–25 min), 34.75% B (25–28 min), 34.75–35.12% B (28-29 min), 35.12% B (29–32 min), and 35.12–100% B (32–35 min). From 200 mg of the CSBt partition, 8.04 mg of compound** 4a**/**4b** and 5.96 mg of compound** 5** were obtained.

### 2.7. Cytotoxicity Assays

*Cell Culture*. The cytotoxicity was evaluated on two cell lines: J774 (macrophage-like cell line, derived from BALB/c murine reticulum cell sarcoma) and WI38 (human normal fibroblast-like lung cell line), using the tetrazolium salt MTT [3-(4,5-dimethylthiazol-2-yl)-2,5-diphenyltetrazolium bromide (Sigma)] colorimetric method, as described by Bero et al. [11]. J774 cells were cultivated in a humidified atmosphere with 5% CO_2_ at 37°C in RPMI 1640 medium (Life Technologies, Paisley, UK) containing 2 mM L-glutamine supplemented with 10% heat-inactivated fetal bovine serum (Life Technologies) and penicillin-streptomycin (100 UI/ml). WI38 cells were cultivated in a humidified atmosphere with 5% CO_2_ at 37°C in DMEM medium (Life Technologies) containing 4 mM L-glutamine, 1 mM sodium pyruvate supplemented with 10% heat-inactivated fetal bovine serum (Life Technologies), and penicillin-streptomycin (100 UI/ml).

*Cytotoxicity Assay*. Briefly, WI38 or J774 cells were seeded in 96-well plates (5000 cells/well/180 *µ*l medium or 2.8 × 10^4^ cells/ml). After 24 h, cells were treated with 20 *μ*l of crude extract and partitions solutions in a fixed concentration of 50 *µ*g/ml to calculate cell viability percentage. When an extract or a partition presents a percentage inferior to 50%, an IC_50_ is calculated with a range of concentration between 0.023 and 50 *µ*g/ml obtained from a 20 mg/ml DMSO stock solution diluted in fresh medium. In the same way, fractions (CSPE1 to CSPE7) obtained from the most active partition (CSPE) and purified compounds were tested at two fixed concentrations of 5 and 20 *µ*g/ml. This time, an IC_50_ is calculated for cell viability percentages inferior to 50% at 20 *µ*g/ml with a range of concentration between 0.01 and 20 *µ*g/ml obtained as cited above. After 72 h, the medium was replaced by 100 *μ*l of a 10% MTT solution (3 mg/ml in PBS) in medium. After 45 min, the medium was removed again and 100 *μ*l of DMSO was added to solubilize formed formazan crystals. Absorbance was recorded at 570 nm and 620 nm. Each sample was tested in eight serial threefold dilutions in 96-well microtiter plates, at least three wells per concentration in duplicate. Camptothecin (Sigma) was used as positive control (concentration range: 25–0.00032 *µ*g/ml). The highest concentration of solvent to which the cells were exposed was 0.25%, which was shown to be nontoxic.

Cell viability% = [*AT*/*ANT*] × 100, with* A*: absorbance,* NT*: control cells, and* T*: treated cells.

### 2.8. Antimicrobial Assay

The antimicrobial activity of crude extract and partitions was evaluated against 36 pathogenic microorganisms (22 Gram-positive bacteria, 13 Gram-negative bacteria, and 1 yeast) according to Mahamodo et al. [12]. The 36 microorganisms were grown overnight at 37°C on sloping Mueller-Hinton (MH) agar medium. Briefly, the bacteria were diluted with Ringer's Cysteine (RC) solution to obtain 10^6^ bacteria/ml. In Petri dishes, 19 ml MH 2 agar was supplemented with 1 ml of stock solution of crude extract or partitions (12.5 mg/ml to 0.39 mg/ml) or antibiotics (gentamicin, vancomycin, and amoxicillin) (28 mg/ml to 6·10^−3^ mg/ml), giving final concentrations from 625 to 19.5 *µ*g/ml for the extracts and 64 to 0.03 *µ*g/ml for antibiotics. Inocula of 10^4^ colony forming units (CFU) were spotted ultimately with a multipoint inoculator (Steers Replicator).

### 2.9. Statistical Analysis

Data were analyzed by GraphPad Prism 7 statistical software and presented as the mean ± standard error of the mean. Activity differences between both cell lines were analyzed by the two-tailed nonparametric Mann–Whitney test to highlight significant toxicity. However, the two-tailed Wilcoxon signed-rank test was used to compare activity (for CSMC, CSEA, CSPE4, and CSPE5) on J774 to the maximum tested value on WI38, 50 or 20 *µ*g/ml, respectively. Statistical significance for all statistical tests was set at *p* < 0.05.

## 3. Results

### 3.1. Cytotoxic Activity of Crude Methanolic Extract and Partitions of* Cirsium scabrum* Leaves

In a previous study [9], we showed that the cytotoxicity of the crude methanolic extract of* C. scabrum* leaves (CSRW) was particularly interesting. It showed, among other plant extracts tested, the highest activity on J774 cell line (IC_50_ = 11.53 *µ*g/ml) and was also quite selective (IC_50_ = 29.89 *µ*g/ml on WI38, selectivity index = 2.6). In this study, our objective is to identify the compounds responsible for this activity. For this purpose, the partitions of* C. scabrum* leaves obtained from the crude methanolic extract by liquid-liquid partitioning (CSPE, CSMC, CSEA, CSBt, and CSW) were also analyzed for their antiproliferative activity on the same cell lines (Table 1). Among all tested partitions, the petroleum ether partition (CSPE) showed the highest activity on J774 with an IC_50_ value (IC_50_ = 12.12 *µ*g/ml) almost similar to that of the crude methanolic extract (CSRW). As CSRW, CSPE was also interesting because it showed some selectivity (IC_50_ = 33.21 *µ*g/ml on WI38, selectivity index = 2.7). A SI value inferior to 2 may indicate a general toxicity of an extract or a pure compound [13]. In our case, we obtained SI superior to 2 for both the crude extract CRSW and the partition CSPE with significant differences (*p* < 0.05) between both cell lines.

### 3.2. Cytotoxic Activity of CSPE Fractions

In view of these results, we followed our bioguided fractionation on the most active partition CSPE. The fractionation of CSPE by silica gel column chromatography produced seven fractions (CSPE1–CSPE7), based on their TLC behaviours, which were analyzed for their antiproliferative activity on both cell lines (Table 2). The highest activity was recorded for the fractions CSPE4 and CSPE5 with IC_50_ values equal to 13.77 and 8.96 *μ*g/ml, respectively. Besides, these fractions did not show any toxicity at 20 *µ*g/ml, the maximum concentration tested on the noncancerous WI38 cell line, and their activity on J774 was significantly different with selectivity indices higher than 1.4 and 2.2, respectively. All other fractions were considered as nonactive (IC_50_ ≥ 20 *µ*g/ml).

### 3.3. Purification of Compounds from CSPE Partition

**Figure 1:**
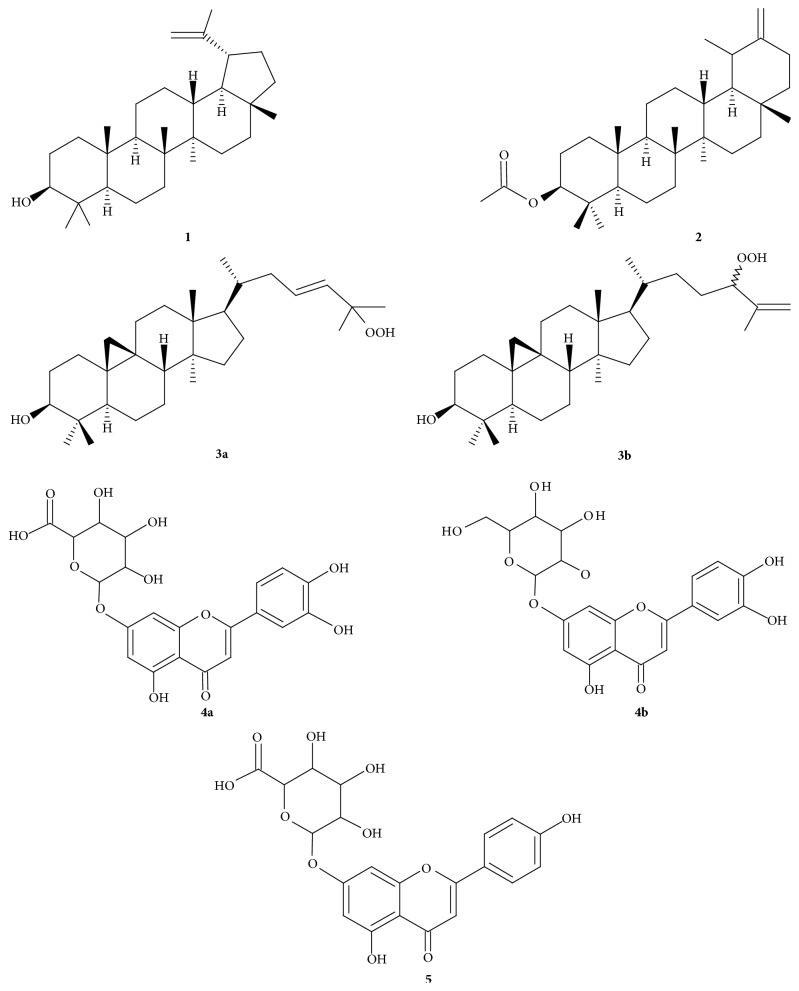
Compounds isolated from* Cirsium scabrum*.

A rapid phytochemical screening revealed that CSPE partition is rich in terpenoids and phytosterols, while the presence of alkaloids, flavonoids, saponins, and tannins was not detected. Four compounds were purified from some CSPE fractions by silica gel column chromatography (CC) or by Centrifugal Partition Chromatography (CPC). All compounds were identified and characterized by their spectroscopic data (^1^H and ^13^C NMR, 2D NMR experiments) and spectra mass data and by comparisons with the literature. Compounds** 1** and** 2** were identified as two common triterpenoids, respectively, lupeol and taraxasterol acetate [14, 15]. Compounds** 3a** and** 3b** were identified as an indissociable (1 : 1) mixture of two hydroperoxide cycloartane-type triterpenoids: 25-hydroperoxycycloart-23-en-3*β*-ol (**3a**) and 24-hydroperoxycycloart-25-en-3*β*-ol (**3b**) [16, 17] (Figure 1).

### 3.4. Cytotoxic Activity of Purified Compounds

The mixture of two cycloartane-type triterpenoids obtained from the most active fractions (combined fractions CSPE4 and CSPE5) was analyzed for their antiproliferative activity on J774 and WI38 cell lines (Table 3). Even if lupeol was isolated directly by CC on silica gel 60 from CSPE and not from CSPE4-5, we tested its activity due to data of the literature [18]. Lupeol exhibited a cytotoxic activity against the J774 cell line with an IC_50_ value similar to that of the CSPE partition (IC_50_ = 12.60 *µ*g/ml) but did not show selectivity against the WI38 cell line (selectivity index = 1.5). On the other side, the mixture of 25-hydroperoxycycloart-23-en-3*β*-ol and 24-hydroperoxycycloart-25-en-3*β*-ol showed the highest activity on J774 cell line (IC_50_ = 1.79 *µ*g/ml) and showed a very interesting selectivity index (IC_50_ = 16.50 *µ*g/ml on WI38, selectivity index = 9.2).

### 3.5. Antimicrobial Activity of Partitions of* Cirsium scabrum* Leaves

In our previous screening conducted on 8 extremophile plants, the crude methanolic extract of* C. scabrum* did not show relevant antimicrobial activity [9]. However, due to the known antimicrobial activity of some* Cirsium* species [7], we decided to analyze again the crude methanolic extract as well as partitions of* C. scabrum* for their antimicrobial activity against a new panel of 36 pathogenic microorganisms including new* Staphylococcus* strains and* Dermabacter hominis* strains.  Table 4 gives MIC (Minimum Inhibitory Concentration) values of the two active partitions: CSEA and CSBt. CSRW, CSPE, CSMC, and CSW were not considered active because they showed MIC values > 1000 *µ*g/ml. CSEA and CSBt showed a moderate antibacterial activity against 6* Staphylococcus aureus* and 2* Dermabacter hominis *strains with higher MIC values for the CSBt partition (312 *µ*g/ml).

### 3.6. Identification of the Major Compounds of the CSBt Partition

A rapid phytochemical screening revealed that CSBt partition is rich in flavonoids and tannins. HPLC analysis of CSBt partition revealed the presence of two major compounds isolated by preparative HPLC. Structural elucidation was achieved based on their spectroscopic data (^1^H and ^13^C NMR, 2D NMR experiments and HRMS) and by comparison with the literature. Compounds** 4a** and** 4b **were identified as a (1 : 1) mixture of luteolin 7-*O*-*β*-D-glucuronide (**4a**) [19] and luteolin 7-*O*-*β*-D-glucoside (**4b**) [20] and compound** 5** was identified as apigenin 7-*O*-*β*-D-glucuronide [21] (Figure 1).

## 4. Discussion

In a previous study conducted on extremophile plants, we analyzed the antiproliferative activity of eight species using the MTT assay on J774 cancerous cell line compared to the one on WI38 noncancerous cell line. We highlighted that the crude methanolic extract of* C. scabrum* leaves (CSRW), a non investigated thistle, showed the highest activity on J774 cell line among other plant extracts tested and a good selectivity index [9]. In this current study, we aimed to investigate the cytotoxic activity of partitions obtained from the extract of* C. scabrum* leaves (CSRW) on the same cell lines. We showed that the petroleum ether partition (CSPE) was the most cytotoxic against the J774 cell line and may contain the compounds mostly responsible for the cytotoxic activity of this species. A bioguided fractionation of CSPE partition allowed us to identify these compounds, a (1 : 1) mixture of two hydroperoxide cycloartane-type triterpenoids: 25-hydroperoxycycloart-23-en-3*β*-ol (**3a**) and 24-hydroperoxycycloart-25-en-3*β*-ol (**3b**). Lee et al. [16] have previously isolated these compounds from the methylene chloride extract of* Cirsium setidens* aerial parts in three steps by successive column chromatography. They demonstrated that both compounds have significant cytotoxic activity against several cultured human cancer cell lines, in particular the 24-hydroperoxycycloart-25-en-3*β*-ol. In our study, both compounds were isolated as a (1 : 1) mixture in one step from the most active combined fractions (CSPE4 and CSPE5) using CPC. The attempts to separate these compounds from each other by column chromatography were not fruitful because they rapidly degrade. Consequently, we tested the antiproliferative activity of the mixture. The cytotoxic activity described in the literature for some* Cirsium* species is not only attributed to cycloartane-type triterpenoids. Other studies highlighted the cytotoxic potential of other types of compounds, for example, caryolane-type sesquiterpenes from* Cirsium souliei* [6] or a nor-*α*-tocopheroid (*α*-tocospiro C) from* Cirsium setosum *[22]. We also isolated from the CSPE partition of* Cirsium scabrum* leaves two common triterpenoids lupeol and taraxasterol acetate. Lupeol and taraxasterol acetate were identified in other* Cirsium *species (*C. canum, C. hypoleucum,* and* C. oleraceum*) [7]. Lupeol is a triterpenoid widely distributed in various botanical families. Several in vitro and preclinical animal studies suggest that lupeol has a wide range of biological activities, including anti-inflammatory, antimicrobial, antiprotozoal, cholesterol lowering, antiproliferative, anti-invasive, and antiangiogenic ones [18]. This natural product is able to modulate different molecular mechanisms contributing to the melanoma development [23, 24]. Taraxasterol acetate is occurring in various* Cirsium* species [7]. For instance, this triterpenoid was isolated from a cytotoxic petroleum ether fraction of* Cirsium setosum *[25] or from* Cirsium* species growing in Turkey [26]. In general, it is quite common in the Asteraceae family [27, 28].

On the other hand, we evaluated the antibacterial activity of the crude methanolic extract and partitions of* Cirsium scabrum* against a panel of 36 Gram-positive and Gram-negative bacteria. The crude extract showed no activity, while the butanol partition CSBt had a moderate action against 6* Staphylococcus aureus* strains and 2* Dermabacter hominis *strains. We isolated the major compounds of the CSBt partition, a (1 : 1) mixture of luteolin 7-*O*-*β*-D-glucuronide and luteolin 7-*O*-*β*-D-glucoside (compounds** 4a** and** 4b**) and apigenin 7-*O*-*β*-D-glucuronide (compound** 5**), as a primary approach to understand the antibacterial potential of* C. scabrum*. To our knowledge, literature does not describe or little describes the antibacterial activity of luteolin 7-*O*-*β*-D-glucuronide and apigenin 7-*O*-*β*-D-glucuronide. However, it is worth mentioning that some plant species containing these two flavones showed antibacterial activity against some Gram-positive and Gram-negative bacteria, including* Staphylococcus aureus* strains [29, 30]. On the other hand, luteolin 7-*O*-*β*-D-glucoside (cynaroside) demonstrated antibacterial activity especially against Gram-negative bacteria [31]. This flavone was already identified in* Cirsium japonicum* [32]. In general, flavonoids are well known to be effective antibacterial substances against a wide array of bacteria, with MIC in the range of 0.06 to 31.3 *µ*g/ml against some Gram-positive bacteria and 0.3 to 39.1 *µ*g/ml against some Gram-negative bacteria, for the ten most potently antibacterial natural flavonoids tested in recent years [33]. As a matter of fact, they are synthesized by plants in response to microbial infection [34]. The mechanism of antimicrobial action of flavonoids seems to be complex as it does not target one specific site of action. In addition to direct and synergistic antibacterial effects, flavonoids interfere with bacterial virulence factors, including quorum-sensing signal receptors, some enzymes, and toxins [33]. The antibacterial activity of apigenin and luteolin against* Staphylococcus aureus* was thoroughly investigated. Apigenin can remarkably decrease at low concentrations the production of *α*-hemolysin, a toxin released by* S. aureus*. Also, apigenin has a therapeutic effect on* S. aureus*-related pneumonia by alleviating injury of the lung tissue and decreasing cytokine levels [35]. Concerning luteolin, Wang and Xie [36] suggested that this flavone could inhibit the activity of DNA topoisomerases I and II, which results in some decrease in the nucleic acid and protein synthesis. A recent study has investigated the mechanism of action of luteolin against methicillin-resistant* Staphylococcus aureus* (MRSA) [37]. The authors showed that luteolin acts in synergy by increasing cytoplasmic membrane permeability and inhibiting ATPase. The study also suggests a direct binding of luteolin with the major constituent of the* S. aureus* cell wall. The antibacterial activity of flavonoids against* D. hominis* was not reported in the literature so far. Besides, little or no information exists on the activity of natural products from plants against this bacteria strain.* D. hominis* is a common colonizer from human skin. It is usually susceptible to vancomycin, teicoplanin, and linezolid [38]. Further phytochemical investigation of the butanol partition of* C. scabrum* would deepen the understanding of its activity against* D. hominis* strains. On the other hand, the antibacterial activity of CSBt partition could be also attributed to the presence of tannins. The antimicrobial activity of tannins is widely investigated. The different mechanisms proposed to explain their mechanism of action include direct action on microbial metabolism but also inhibition of extracellular microbial enzymes or deprivation of the substrates required for microbial growth [39]. Nazaruk et al. [40] reported the presence of tannins in aqueous extracts from leaves of several* Cirsium* species. They showed that most of these extracts had antibacterial potential. Although they suggested that the content of small-molecular phenolic compounds had greater influence on the activity of extracts than tannins, consideration will be given to investigate more the antibacterial effect of the CSBt partition from* C. scabrum*.

## 5. Conclusion

A phytochemical and biological study was carried out for the first time on* Cirsium scabrum*. The study revealed that the crude methanolic extract of the leaves and more precisely the petroleum ether partition (CSPE) showed an interesting and quite selective cytotoxic potential against the J774 cell line. A mixture of two hydroperoxide cycloartane-type triterpenoids was isolated and identified as the secondary metabolites responsible for this activity. Two other triterpenoids, lupeol and taraxasterol acetate, were also isolated from the CSPE partition, strengthening our knowledge about the chemical composition of* Cirsium scabrum*. We also focused our study on the antibacterial potential of this species. The butanol partition of* C. scabrum* leaves (CSBt) showed a moderate activity against 6* Staphylococcus aureus* strains and 2* Dermabacter hominis *strains. The two major compounds of CSBt partition were identified as glycosides of flavones, suggesting that they might be responsible for this activity, as well as the presence of tannins.

## Figures and Tables

**Table 1:** In vitro cytotoxicity of the crude extract and partitions of *C. scabrum* leaves.

Crude extract and partitions	WI38 IC_50_ (*µ*g/ml)	J774 IC_50_ (*µ*g/ml)	SI (IC_50 WI38_/IC_50_ J_774_)
CSRW	29.89 ± 2.38	11.53 ± 1.07	2.6^*∗∗∗*^
CSPE	33.21 ± 2.22	12.12 ± 0.34	2.7^*∗∗∗*^
CSMC	>50	48.13 ± 1.87	>1
CSEA	>50	40.97 ± 2.91	>1.2^*∗*^
CSBt	>50	>50	>1
CSW	>50	>50	>1
Camptothecin	0.175 ± 0.029	0.011 ± 0.002	15^*∗∗∗*^

CSRW: *C. scabrum* leaves crude extract; CSPE: *C. scabrum* leaves petroleum ether partition; CSMC: *C. scabrum* leaves methylene chloride partition; CSEA: *C. scabrum* leaves ethyl acetate partition; CSBt: *C. scabrum* leaves butanol partition; CSW: *C. scabrum* leaves water partition; camptothecin: positive control; results are expressed as mean ± standard error of the mean (SEM) from six determinations and activity differences between cell lines are statistically defined for each partition with the Mann–Whitney or Wilcoxon signed-rank tests for comparison to the highest tested concentration (*p* < 0.05^*∗*^, *p* < 0.005^*∗∗∗*^).

**Table 2:** In vitro cytotoxicity of CSPE fractions.

CSPE fractions	WI38 IC_50_ (*µ*g/ml)	J774 IC_50_ (*µ*g/ml)	SI (IC_50 WI38_/IC_50_ J_774_)
CSPE1	>20	>20	>1
CSPE2	>20	>20	>1
CSPE3	>20	>20	>1
CSPE4	>20	13.77 ± 1.26	>1.4^*∗*^
CSPE5	>20	8.96 ± 1.09	>2.2^*∗*^
CSPE6	>20	>20	>1
CSPE7	>20	>20	>1
Camptothecin	0.175 ± 0.029	0.011 ± 0.002	15^*∗∗∗*^

CSPE1–CSPE7: fractions of CSPE; camptothecin: positive control; results are expressed as mean ± standard error of the mean (SEM) from six determinations and activity differences between cell lines are statistically defined for each fraction with the Mann–Whitney or Wilcoxon signed-rank tests for comparison to the highest tested concentration (*p* < 0.05^*∗*^, *p* < 0.005^*∗∗∗*^).

**Table 3:** In vitro cytotoxicity of isolated compounds from the combined fractions CSPE4/CSPE5.

	WI38 IC_50_ (*µ*g/ml)	J774 IC_50_ (*µ*g/ml)	IS (IC_50 WI38_/IC_50_ J_774_)
Compound 1	19.24 ± 0.55 (4.51 *µ*M)	12.60 ± 0.23 (2.96 *µ*M)	1.5^*∗∗∗*^
Mixture of compounds 3a/3b	16.50 ± 0.24 (3.6 *µ*M)	1.79 ± 0.03 (0.39 *µ*M)	9.2^*∗∗∗*^
Camptothecin	0.175 ± 0.08 (0.05 *µ*M)	0.011 ± 0.004 (0.0031 *µ*M)	15^*∗∗∗*^

Compound 1: lupeol; mixture of compounds 3a/3b: 25-hydroperoxycycloart-23-en-3*β*-ol and 24-hydroperoxycycloart-25-en-3*β*-ol; camptothecin: positive control; results are expressed as mean ± standard error of the mean (SEM) of six determinations and activity differences between cell lines are statistically defined for each compound with the Mann–Whitney or Wilcoxon signed-rank tests for comparison to the highest tested concentration (*p* < 0.005^*∗∗∗*^).

**Table 4:** Antimicrobial activity of active partitions of *C. scabrum *leaves.

	Active partitions MIC values (*µ*g/ml)		Reference antibiotics MIC values (*µ*g/ml)		
	CSBt	CSEA	Amox	Gent	Vanc
Gram – bacteria					

*Escherichia coli* T20A2	NA	NA	S	R	**R**
*Pseudomonas aeruginosa *T41	NA	NA	R	S	R
*Pseudomonas aeruginosa *ATCC9027	NA	NA	R	S	R

Gram + bacteria					

*Dermabacter hominis *T47A7	**312**	**625**	R	S	S
*Dermabacter hominis *T49B5	**312**	**625**	S	S	S
*Corynebacterium striatum *T40A3	NA	NA	R	R	S
*Corynebacterium striatum *T46C1	NA	NA	S	S	S
*Enterococcus faecalis *T37B1	NA	NA	S	S	S
*Enterococcus faecalis *T47A16	NA	NA	R	S	S
*Streptococcus agalactiae* T53A4	NA	NA	R	R	R
*Staphylococcus aureus *T2510	**312**	**625**	R	S	S
*Staphylococcus aureus *T281	NA	NA	R	S	S
*Staphylococcus aureus *8143	**312**	**625**	S	S	S
*Staphylococcus aureus *8148	**312**	**625**	R	S	S
*Staphylococcus aureus *8142	NA	NA	R	S	S
*Staphylococcus aureus *T61	NA	NA	R	S	S
*Staphylococcus aureus *T21	**312**	**625**	R	S	S
*Staphylococcus aureus *T11	NA	NA	R	S	S
*Staphylococcus aureus *T306	**312**	**625**	S	S	S
*Staphylococcus aureus *T26A4	**625**	**625**	R	S	S
*Staphylococcus epidermidis *T151	NA	NA	R	R	S
*Staphylococcus epidermidis *T19A1	NA	NA	S	S	R
*Staphylococcus capitis *T21A3	NA	NA	S	S	S
*Staphylococcus capitis *T29A2	NA	NA	S	S	S
*Staphylococcus pettenkoferi *T282	NA	NA	S	S	S
*Staphylococcus pettenkoferi *T33	NA	NA	R	S	S
*Staphylococcus saprophyticus 8237 *	NA	NA	S	S	S
*Staphylococcus lugdunensis *T36A1	NA	NA	S	S	S
*Staphylococcus lugdunensis *T47B2	NA	NA	S	S	S

MIC (*µ*g/ml) of positive controls: Amox (amoxicillin), S: ≤4, R: >16, Gent (gentamicin), S: ≤4, R: >8; Vanc (vancomycin), S: ≤4, R: >16; MIC (*µ*g/ml) of partitions (CSBt: *C. scabrum* leaves butanol partition; CSEA: *C. scabrum* leaves ethyl acetate partition): MIC < 100 *µ*g/ml: high activity; 100 *µ*g/ml < MIC < 500 *µ*g/ml: moderate activity; 500 *µ*g/ml < MIC < 1000 *µ*g/ml: low activity; MIC > 1000 *µ*g/ml: NA = inactive.
